# A Screening Approach to Assess the Impact of Various Commercial Sources of Crude Marine λ-Carrageenan on the Production of Oligosaccharides with Anti-heparanase and Anti-migratory Activities

**DOI:** 10.3390/md21050295

**Published:** 2023-05-11

**Authors:** Chanez Manseur, Hugo Groult, Manon Porta, Pierre-Edouard Bodet, Rachida Mersni-Achour, Raphaëlle Petit, Samir Ali-Moussa, Benjamin Musnier, Didier Le Cerf, Tony Varacavoudin, Oualid Haddad, Angela Sutton, Cíntia Emi Yanaguibashi Leal, Edilson Beserra Alencar-Filho, Jean-Marie Piot, Nicolas Bridiau, Thierry Maugard, Ingrid Fruitier-Arnaudin

**Affiliations:** 1 UMR CNRS 7266, LIENSs Laboratory, La Rochelle University, 17000 La Rochelle, France; chanez.manseur@univ-lr.fr (C.M.); manon.porta@univ-lr.fr (M.P.); pierreedouard.bodet@univ-lr.fr (P.-E.B.); rachida.mersni@univ-lr.fr (R.M.-A.); rpetit85@gmail.com (R.P.); alimoussasamir4@gmail.com (S.A.-M.); benjamin.musnier@univ-lr.fr (B.M.); jean-marie.piot@univ-lr.fr (J.-M.P.); nicolas.bridiau@univ-lr.fr (N.B.); thierry.maugard@univ-lr.fr (T.M.); 2 Sciences & Technic Faculty, Univ Rouen Normandie, INSA Rouen Normandie, CNRS, PBS UMR 6270, 76000 Rouen, France; didier.lecerf@univ-rouen.fr (D.L.C.); tony.varacavoudin@univ-rouen.fr (T.V.); 3 Inserm U1148, Laboratory for Vascular Translational Science, UFR SMBH, Université Paris 13, Sorbonne Paris Cité, Groupe Biothérapies et Glycoconjugués, 93000 Bobigny, France; haddad.oualid@univ-paris13.fr (O.H.); angela.sutton@univ-paris13.fr (A.S.); 4 College of Pharmaceutical Sciences, Federal University of Vale do São Francisco (UNIVASF), Petrolina 56304-205, PE, Brazil; cintia.leal@discente.univasf.edu.br (C.E.Y.L.); edilson.beserra@univasf.edu.br (E.B.A.-F.)

**Keywords:** marine algae, lambda-carrageenan, oligosaccharides, heparanase, migration, anticancer agents

## Abstract

Oligosaccharides derived from λ-carrageenan (λ-COs) are gaining interest in the cancer field. They have been recently reported to regulate heparanase (HPSE) activity, a protumor enzyme involved in cancer cell migration and invasion, making them very promising molecules for new therapeutic applications. However, one of the specific features of commercial λ-carrageenan (λ-CAR) is that they are heterogeneous mixtures of different CAR families, and are named according to the thickening-purpose final-product viscosity which does not reflect the real composition. Consequently, this can limit their use in a clinical applications. To address this issue, six commercial λ-CARs were compared and differences in their physiochemical properties were analyzed and shown. Then, a H_2_O_2_-assisted depolymerization was applied to each commercial source, and number- and weight-averaged molar masses (M_n_ and M_w_) and sulfation degree (DS) of the λ-COs produced over time were determined. By adjusting the depolymerization time for each product, almost comparable λ-CO formulations could be obtained in terms of molar masses and DS, which ranged within previously reported values suitable for antitumor properties. However, when the anti-HPSE activity of these new λ-COs was screened, small changes that could not be attributed only to their small length or DS changes between them were found, suggesting a role of other features, such as differences in the initial mixture composition. Further structural MS and NMR analysis revealed qualitative and semi-quantitative differences between the molecular species, especially in the proportion of the anti-HPSE λ-type, other CARs types and adjuvants, and it also showed that H_2_O_2_-based hydrolysis induced sugar degradation. Finally, when the effects of λ-COs were assessed in an in vitro migration cell-based model, they seemed more related to the proportion of other CAR types in the formulation than to their λ-type-dependent anti-HPSE activity.

## 1. Introduction

The marine ecosystem is taking a real interest in the research that aims to identify new natural anticancer molecules for targeted therapies [1]. Among them, carrageenans of lambda type (λ-CARs) are among the most promising bioactive marine carbohydrates [2]. They are high-molar-mass (100–1000 kDa) linear-sulfated polysaccharides (SPs) extracted from different species of red seaweeds *(Gigartinales Rhodophyta)* [3]. Their backbone is mainly composed of 3-linked β-d-galactopyranose (G-units) sulfated in position 2- and 4-linked α-d-galactopyranose (d-units) sulfated in position 2 and 6 forming the disaccharide repeating unit of CARs [4]. In addition to their significant use as versatile ingredients in food preparation and cosmetics as thickening, stabilizing, or gelling agents, the biological properties of these polygalactans have also been the subject of particular attention in pharmaceutical formulations and experimental medicine [5,6]. λ-CARs have a wide range of bioactivities, including anticoagulant, immunomodulatory, antiviral, and antitumor effects [4,5,7,8,9,10,11]. Nevertheless, they have some drawbacks that can limit their medical application. In fact, in their native form, they are characterized by low solubility, high viscosity, and poor bioavailability, making their intravenous use almost impossible [12]. Moreover, their broad range of activities can include adverse effects. For instance, they are known to have potent proinflammatory and anticoagulant activities [13,14,15]. This is illustrated by their common use in preclinical studies to induce acute inflammation in animal models when assessing anti-inflammatory drugs [14,16,17].

To counter these limitations, the development of low molar mass (LMW) fractions called oligosaccharides (OSs) is unavoidable [18,19]. Production of OSs derived from λ-CARs (abbreviated as λ-COs) has been assessed using different methods, including chemical, physical, and enzymatic degradations [19,20,21], sometimes associated with chemical post-modifications. Therefore, many λ-COs have been successfully prepared in order to have physicochemical properties suitable for in vivo applications and a better specificity for bioactivity of interest compared with their native forms [19,22,23]. In this study, we focus on investigating the anticancer activity of λ-CARs and their λ-COs derivatives. Indeed, several studies have described promising effects, especially through the stimulation of the immune response [23,24,25,26,27,28], radiotherapy effects enhancement [29], the induction of cancer cells’ apoptosis [30,31,32,33,34], interaction with growth factors, or disruption of endothelial cells metabolisms in the extracellular matrix (ECM) [21,35,36,37]. One of the most promising antitumor mechanisms of λ-COs reported involves the inhibition of heparanase (HPSE) activity [38], an enzyme strongly implicated in tumor progression and overexpressed in most cancers. HPSE is the sole endoglycosidase able to cleave heparan sulfate (HS) chains, an important component of the ECM in the tumor microenvironment. Its enzymatic action leads to ECM remodeling that promotes cell invasion and to a release of HS-binding proteins (HSBPs) and cytokines that induce proliferative, angiogenic, and inflammatory processes [39,40]. Many studies have focused on the inhibition of HPSE activity by producing sulfated-based carbohydrate biomolecules that mimic the HS natural substrate [41]. Following this strategy, previous works have described in vitro promising anti-angiogenic activity of λ-COs [42], and anti-invasiveness effects [43,44,45,46] on different cancerous cell lines, both partially mediated by the inhibition of HPSE [47,48].

Despite these promising results, the clinical maturation of λ-COs to medically valorize these algal PSs still faces challenges. In fact, these candidates are formulations containing different biomolecules with variable dispersity (especially in length/molar mass and sulfation pattern) that should meet the Good Medical Practice standards for pharmaceutical development. Recent studies on OSs derived from marine SPs have shown that advances in purification processes are paving the way for the reproducible production of clinical-grade batches [49]. However, this can be fastidious, especially on an industrial scale, and efforts are still needed to control the heterogeneity of the λ-COs produced before any purification steps. The latter can come from the depolymerization method used, which may generate disparities in some cases (e.g., “harsh” H_2_O_2_-based radical depolymerization is not selective and may affect the carbohydrate backbone), and more likely, can come from the starting raw material of native polysaccharides that undergo the depolymerization reaction [50,51]. Indeed, as for any marine polysaccharides, λ-CAR composition and structure depend on the algal source, cell cycle, life stage, growth, environment, and the first industrial extraction method applied [52]. In addition, λ-CARs present a very specific particularity. They are produced by the tetrasporophyte form of the algae, whereas gametophytes mostly synthesize hybrid kappa (κ) and iota (ι) CARs [53]. The tetrasporophytes and gametophytes are difficult to distinguish macroscopically, so they are often collected together, and therefore, industrial λ-CARs are a mixture of miscellaneous CARs. In addition, additives or other types of carrageenan may be added in order to standardize the texture functionality of products principally intended for agricultural or cosmetic industries. Thus, the ‘’λ’’ appellation of commercial products is based on the thickening behavior of the mixtures obtained, rather than the molecular structure, leading to the variable proportion of true theoretical λ-disaccharide units (λ-diads) [54].

With this background, it appears essential to investigate the influence of all these features on the perspective of clinical-grade λ-COs production with anti-HPSE properties. In this study, six commercial native λ-CAR products were compared, and their initial characterization already highlighted distinct traits. The impact of these differences was assessed on three basic features of λ-COs produced over time by a radical-H_2_O_2_-based method [44]), i.e., the molar masses (M_n_, M_w_), the polydispersity index (Ð), and the degree of sulfation (DS). Next, an in vitro biofunctional monitoring was also performed, looking at the λ-COs abilities to inhibit HPSE according to the molar masses and DS. Comparative mass spectrometry (MS) and ^1^H nuclear magnetic resonance (NMR) analyses were also performed to elucidate the composition and molecular structures of the species contained in the different formulations obtained from each supplier and their eventual impact on the anti-HPSE activities, as well as the influence of the structural changes resulting from the chemical H_2_O_2_-based depolymerization method. Finally, selected λ-COs from each supplier were assessed by a migration cell-based assay using hepatocyte carcinoma Huh7 known to overexpressed HPSE; this was considered to be another potential framework for studying the possible influence on the λ-CARs sources.

## 2. Results and Discussion

### 2.1. Physicochemical and Structural Characterizations of Native λ-CARs

**Figure 1:**
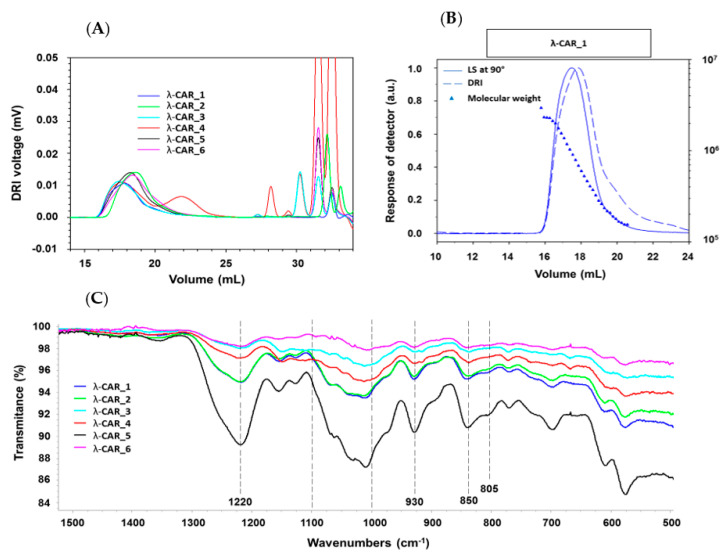
(**A**) DRI chromatograms. (**B**) Example of cumulative molar mass obtained from the HPSEC-MALS analyses of λ-CAR_1 and (**C**) FTIR spectra of the six native commercial λ-CARs.

**Figure A1:**
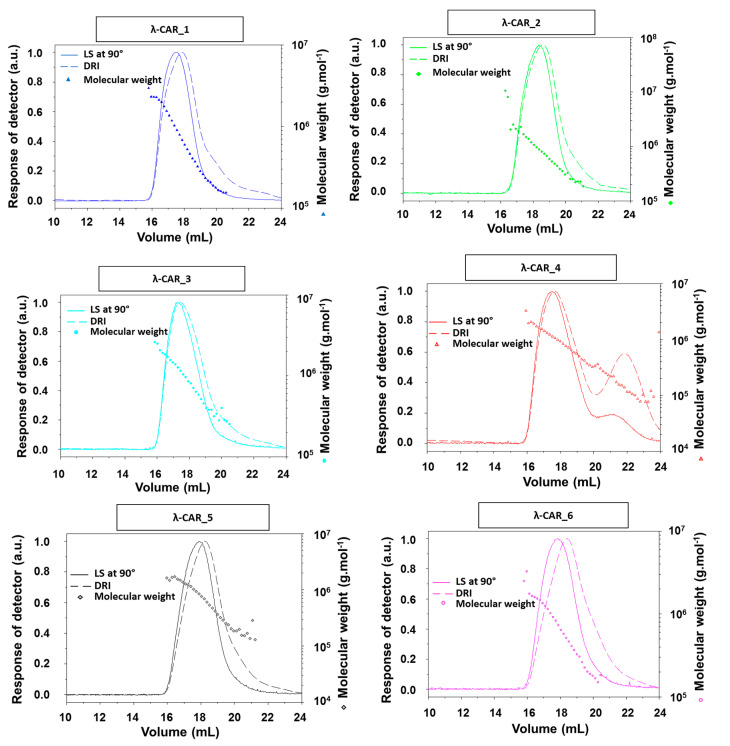
Cumulative molar mass of HPSEC-MALS analysis of native λ-CARs.

The physicochemical properties of the six native commercial λ-CARs (randomly named from 1 to 6) were first determined based on their M_n_, M_w_, Ð, and DS estimations (Table 1). The calculation of the absolute molar mass relying on the HPSEC-MALS analyses showed clear differences in M_n_ and M_w_ of λ-CARs, which could range, respectively, from 597 and 964 kDa for λ-CAR from supplier_3 to 215 and 762 kDa for λ-CAR from supplier_1 (Table 1 and Appendix A, Figure A1). There were also significant differences in Ð. In particular, additional peaks were detected using a DRI in the 27–35 mL region of the chromatograms, just before the solvent peak, showing the presence of additional LMW species in the mixtures (Figure 1A). These species were more pronounced in the λ-CAR from supplier_4, which already showed two distinct populations in the sample signal (at 18 and 22 mL). Then, the ICP-MS analyses showed that the λ-CAR from suppliers_1,2,5, and _6 were more sulfated (26.0%, 27.0%, 28.5%, and 28.9%, respectively) than the λ-CAR from supplier_3 and _4 (21.4% and 21.9%, respectively). These results confirmed the presence of dissimilarities in terms of M_n_, DS, and Ð between the native λ-CARs depending on the supplier.

The ICP-MS analyses also revealed differences in the basic elemental composition of different raw materials, especially in terms of iron, magnesium, and manganese content (Appendix A, Table A1). Finally, a first basic overview of the structural characterization was achieved by FTIR. A typical absorption corresponding to SP was observed at about 1250 cm^−1^, corresponding to the sulfate ester groups present in six commercial native λ-CARs. Marked broad-absorption bands in the 1000–1100 cm^−1^ region were also observed, corresponding to representative chemical functions of these polysaccharides, as summarized by Fernàndez et al. [55]. When analyzing more thoroughly the vibrations characterizing the CARs families, all the observed spectra presented vibrations at about 820–830 cm^−1^, corresponding, respectively, to 2- and 6-sulfate galactopyranose of the λ-CAR type. However, a weak signal at about 805 cm^−1^, corresponding to 3,6-anhydrogalactose-2-sulphate present in the ι-family was also detected. In addition, strong bands at 850 and 930 cm^−1^ were also observed, corresponding to 3,6-anhydro-d-galactose and 4-sulfate d-galactose present in both κ and ι-CARs types, respectively. Therefore, FTIR analysis confirmed the presence of κ and ι-CARs sequences in the six commercial native λ-CARs.Overall, these first results confirmed that commercial λ-CARs can present differences in their physicochemical features. Such a variation is well known for polysaccharides extracted from natural resources, the features of which will depend on the algal source, growth/environmental conditions, or industrial steps required for their extraction [52,56,57]. Interestingly, the FTIR analysis also confirmed the additional facet specific to the commercial λ-CARs that contain various proportions of other CAR types (κ/ι) depending on the reproductive cycle at which algae were collected [53].

### 2.2. Native λ-CARs Depolymerization for λ-COs Production

**Figure 2:**
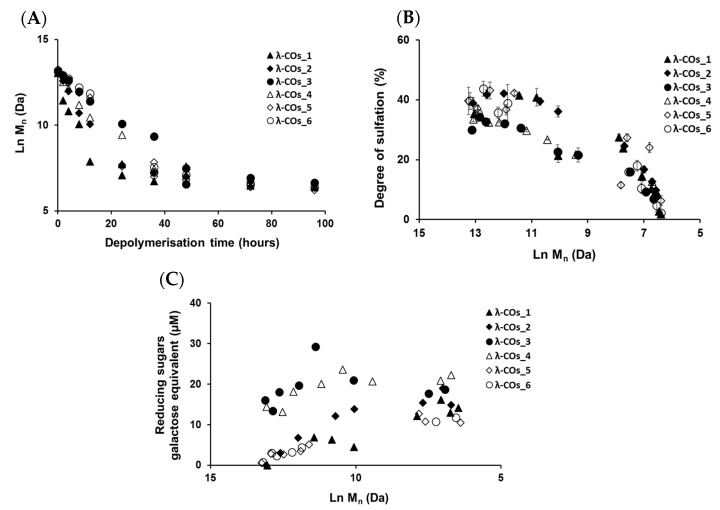
(**A**) Evolution of the number-averaged molar mass (M_n_) of the λ-COs obtained by H_2_O_2_-based depolymerization from the six commercial λ-CARs. (**B**) Effect of depolymerization on the degree of sulfation (DS) of the λ-COs generated from each commercial λ-CAR. (**C**) Reducing sugar quantification over M_n_ decrease.

**Figure A2:**
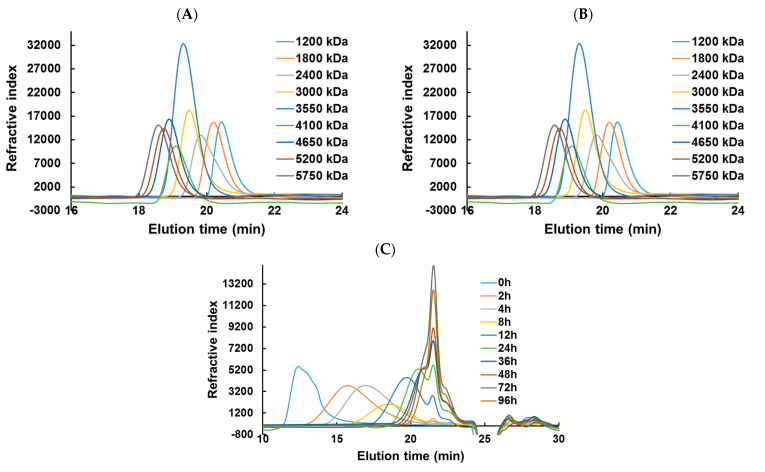
HPSEC-DRI analysis of pullulan (**A**), heparin (**B**) standards, and the produced λ-COs_1 along the time (**C**). The SEC separation was performed on a TSK-GEL G4000PW column in series with a TSK-GEL G3000PW XL at a flow rate of 0.8 mL/min using 0.1 M sodium nitrate (NaNO_3_) as the eluent.

λ-COs were generated from the commercial native λ-CARs using a H_2_O_2_-based radical hydrolysis, one of the most commonly used methods for CAR OSs production [19]. Hydrolysis was performed at 60 °C with an H_2_O_2_/λ-CARs ratio (*w/w*) of 1.5, two parameters previously described as suitable for λ-COs production [44]. HPSEC-DRI analyses allowed determining the molar masses (M_n_, M_w_) of the λ-COs produced using a calibration curve of pullulan standards for species above 10 kDa and a calibration curve of heparin standards for species below 10 kDa (Table 1 and Appendix A, Figure A2). Moreover, the M_n_ of selected λ-COs was also calculated by HPSEC-MALS (Appendix A, Figure A5, Table A2), and showed that the M_n_ values were close to those of heparin equivalent, confirming the accuracy of using heparin standards to estimate the M_n_ and M_w_ of LMW OSs. As already shown in [44], a decrease in M_n_ was observed over time, confirming the depolymerization method efficiency (Figure 2A). Clear differences in the M_n_ were observed between the λ-COs of each commercial native λ-CAR before 24 h depolymerization time, which could be explained by the differences in the M_n_ observed between the different native λ-CARs (see above). In fact, a slower depolymerization kinetic was observed for λ-CAR from supplier_3, which showed the highest M_n_ of 597 kDa compared with the λ-CAR from supplier_1 that was depolymerized more rapidly than all other native λ-CARs and presented the lowest M_n_ of 215 kDa. However, this depolymerization kinetics of native λ-CARs was not the same for all the commercial products, suggesting the influence of other parameters, such as a structural conformation, more resistant to hydrolysis [4]. Interestingly, after 24 h depolymerization, the M_n_ values of all generated λ-COs started to become closer over time, suggesting more similar structural LMW patterns, and thus a similar efficiency in the H_2_O_2_-based hydrolysis.

The DS of all generated λ-COs was also measured over time during depolymerization (Figure 2B). A slow decrease was observed when the M_n_ was > 20 kDa, whereas it became faster after this M_n_ threshold, while the depolymerization reaction slowed down, as shown in Figure 2A and in our previous work [44]. This desulfation behavior at the beginning of the reaction could be explained by the complex tridimensional structure of native λ-CARs [4], which could limit the exposure of sulfate groups to the H_2_O_2_ depolymerization agent and thus, lead to a slow desulfation at the beginning of the reaction. However, this structure was removed through depolymerization, and the sulfate groups were increasingly exposed to the H_2_O_2_ agent, increasing the kinetics of desulfation (Figure 2B). It has also been previously suggested that differences in resistance to hydrolysis, depending on the position of the three sulfate groups that theoretically compose the λ-diads, could explain this observation [44]. Important differences in DS between all the λ-COs were mainly found when the M_n_ was above > 10 kDa and were consistent with the variations observed between the native products: i.e., the higher the DS of the native product, the higher the DS of the resultant λ-COs compared with the other. Again, the DS appeared more similar between shorter λ-COs. These two observations indicated that even if the initial products showed differences, the generated λ-COs < 10 kDa demonstrated more homogeneous M_n_ and DS regardless of the origin of the native λ-CARs and provided an adjustment of the depolymerization time according to each brand (Figure 2A,B and Table 2). 

To complete this physicochemical characterization, the levels of reducing sugars were measured in the solutions during the depolymerization process (Figure 2C). An overall trend of increasing concentrations of reducing ends (expressed in galactose equivalent) was observed, which showed that the hydrolysis of glycosidic bonds resulting from the depolymerization reaction produced new reducing terminal galactoses over M_n_ reduction. However, after the reaction time corresponding approximatively to the production of 10 kDa OSs, the concentration of reducing sugars began surprisingly to decrease even though the chain length continued to be reduced. This suggested a possible change in terminal monosaccharides that will be further studied (see analyses of the selected λ-COs by MS and NMR). It is noteworthy that the level of reducing ends was especially high at the beginning of depolymerization for OSs originating from supplier_3 and supplier_4, suggesting the presence of small reducing sugars in the mixtures, such as glucose, which is often used as an adjuvant [58].

Finally, the reproducibility of the H_2_O_2_-based depolymerization was assessed because of its importance for large-scale production of bioactive λ-COs. In this regard, the reaction was repeated using the same depolymerization times on all commercial λ-CARs (Table 2, dep (1) and dep (2)). The M_n_ and DS were calculated and differences were observed between both depolymerization reactions (dep1 and dep2). For example, for the λ-CAR from supplier_1, a λ-CO with an M_n_ of 1.2 kDa and DS of 14% was obtained after a first depolymerization for 24h, whereas a λ-CO with higher M_n_ and DS of 2.3 kDa and 24%, respectively, was obtained after the second depolymerization. This result could be explained by the experimental setup, which was not implemented in bioreactors where the conditions are tightly controlled so that some temperature fluctuation was possible. Additionally, λ-CARs were subjected to conditions close to the solubility limit at the beginning of the reaction, resulting in a heterogeneous medium, which could also explain some variations [4]. However, it should be noted that, with few exceptions, the M_n_ and DS of each OS remained correlated regardless of their origin; in other words, one given M_n_ is associated with a specific DS. This suggests that with a real-time adjustment of the reaction (looking “live” at M_n_ and DS parameters), it is possible to obtain similar formulations with identified characteristics. Such “live” controls are quite common for the preparation of LMW PS, e.g., a biological control based on anti-Xa activities is performed for the industrial production of standard batches of LMW heparins [59].

### 2.3. Study of the Inhibition of Heparanase Activity by λ-Cos

**Figure 3:**
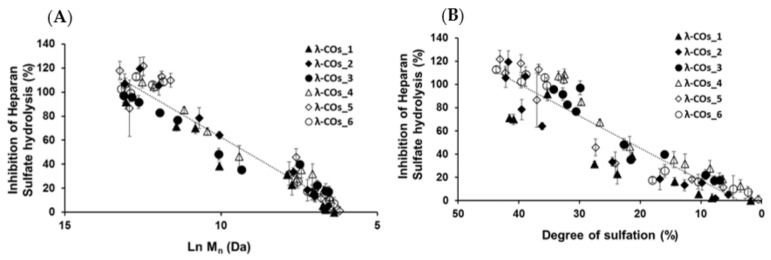
(**A**) Influence of the number-averaged molar mass (M_n_) of λ-COs on the anti-heparanase (HPSE) activity at 2.5 µg·mL^−1^. (**B**) Importance of the degree of sulfation (DS) on the inhibition of HPSE activity.

**Figure A3:**
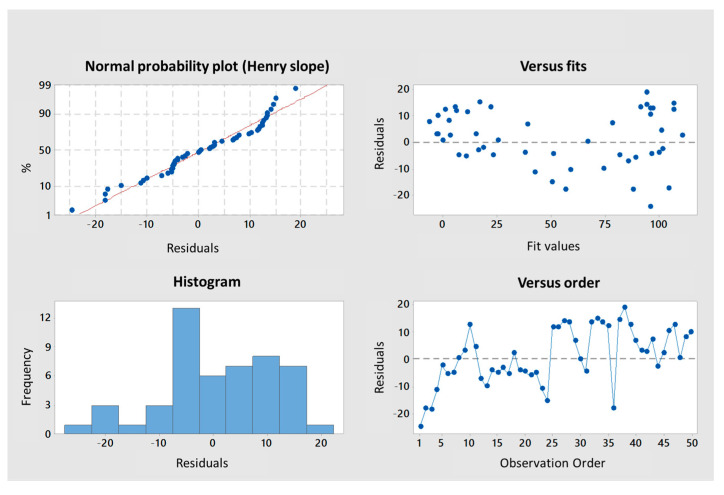
Results of multiple linear regression statistical analysis on the λ-COs library from each supplier.

Next, we achieved biofunctional monitoring across the λ-COs chemical library of different anti-HPSE activities for which λ-COs are considered promising [38,42,43,44,45,46]. Thus, this biomonitoring of λ-COs was assessed at a concentration of 2.5 µg·mL^−1^, suitable to encompass inhibition concentrations of all the λ-COs. As expected, anti-HPSE activity decreased according to the M_n_ and DS reductions throughout depolymerization (Figure 3A,B), regardless of the starting material. Taken independently, the two variables Ln (M_n_) showed a complete linear Pearson correlation with the inhibition of the HPSE activity, with *r*^2^ of 0.962 and 0.929 (*p*-values < 1 × 10^−5^), respectively. A multiple linear regression based on the least squares method was then performed using the two variables together. The model obtained was consistent (Appendix A, Figure A3), explaining 97.6% of the variance of the system (Table 3). Both predictors were significant with coefficients of 10.04 ± 1.26 and 1.189 ± 0.162 for M_n_ and DS, respectively. As already reported, because H_2_O_2_ both depolymerizes and causes desulfation, it is difficult to distinguish the more important parameter for the anti-HPSE activity with this method [44]. On the one hand, standardized coded values after normalization by the averages and scales indicated that DS might be slightly more influential than the chain length. The presence of sulfate groups is commonly described as very favorable for the conception of OS-based HPSE inhibitors [40,41]. On the other hand, at the beginning of the depolymerization (when Ln(Mn) went from 15 to around 10 KDa), although there is only a slight decrease in DS (Figure 2B), anti-HPSE activity still decreased substantially (Figure 3A). In addition, a previous study already showed a general trend of reduction in HPSE inhibition along with λ-COs shortening [45]. Broadening the discussion, many studies also described an increase in antitumor or immune-stimulating activities for LMW λ-CAR derivatives that might be partially mediated by HPSE modulation [24,28]. Overall, this general trend observed for all λ-CARs was expected, but we wanted to further analyze the importance of the raw material in the production of bioactive λ-COs with anti-HPSE activity, which was not yet investigated. 

**Figure A4:**
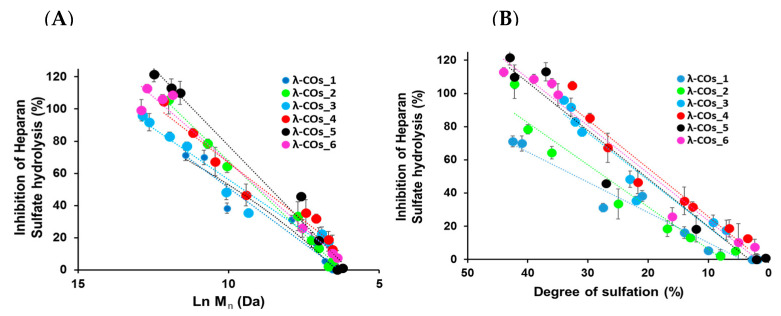
Single linear regression statistical analysis on the M_n_ (**A**) and DS (**B**) relation with anti-HPSE activity of the λ-COs separated by suppliers.

Similar independent and multiple linear regression analyses were performed, and the λ-COs were grouped and analyzed according to their commercial origin. The R² values, reflecting the variance in the multivariate system, were higher than the one obtained with the analysis of all the generated λ-COs, showing good agreement between the HPSE inhibition values calculated for λ-COs issued from the same supplier. Interestingly, in the independent individual analysis, differences were found between the coefficients of the Ln (M_n_), which could range from 12.75 for λ-COs from supplier_3 to 18.49 for λ-COs from supplier_2, as well as between the coefficients of the DS, which could range from 1.82 for λ-COs from supplier_1 to 3.14 for λ-COs from supplier_4 (Appendix A, Figure A4). These data suggested that the ability to inhibit HPSE activity is not only dependent on the M_n_ and DS but can vary with the starting material and slightly differ in the case of λ-COs with the same M_n_ and DS. However, these statistical values should be interpreted with caution given the smaller populations used when separating the λ-COs library according to the commercial suppliers (as evidenced by the loss of significance of several *p*-values) and the assumption of linearity across the scale, which could not be the case for the extremely small and highly desulfated OSs.

To support this hypothesis regarding the role of parameters other than M_n_ and DS influencing the inhibition of HPSE activity and to deepen the biological evaluation, six λ-CO from each λ-CAR supplier with close (although slightly different) M_n_, M_w_, and DS were selected. Instead of assessing the anti-HPSE activity at a single concentration, the half-maximal concentrations (IC_50_) were calculated for each candidate (Table 4 and Appendix A, Figure A6). The results were consistent and similar to those reported previously under the same experimental conditions [43]. In this case, moderate to strong Pearson correlations but with lower significance were found between the calculated Ln (M_n_), DS and IC_50_ variables: −0.887 (*p*-value 0.003) and −0.663 (*p*-value 0.073), respectively. This reinforces the hypothesis that slight variations in M_n_, M_w_, and DS could not fully explain the differences in anti-HPSE activities observed for these six λ-COs. Thus, while this bioactivity is obviously related to M_n_ and DS, these observations imply the possibility that it could also be “supplier-dependent”, i.e., related to potential structural or composition differences (carrageenan types, salt content, and additives) between the different commercial products. When IC_50_ was calculated directly for the native λ-CAR obtained from each supplier (Table 4 and Appendix A, Figure A6), no significant correlations at all were found (*p*-value up to 0.3 for IC_50_ vs Ln (Mn) or DS) 

### 2.4. MS and NMR Analyses of Selected λ-Cos

**Figure 4:**
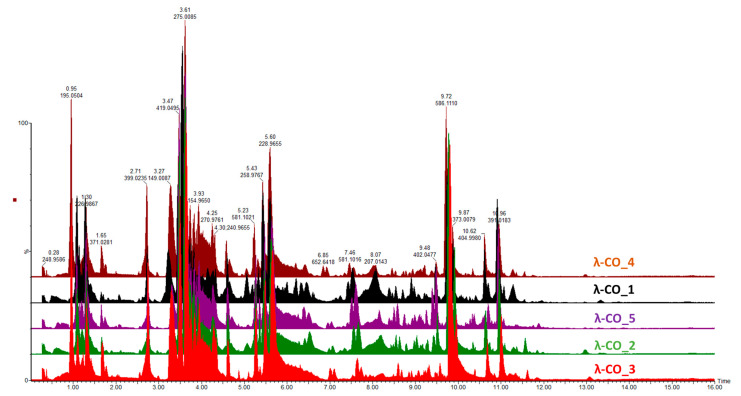
MS chromatographic profiles obtained in negative polarity of a selected λ-CO from each supplier.

Given the previous results, it was important to go further in detecting species constituting the λ-COs formulations and to advance the understanding of their first basic structural determination. The first set of analyses performed on the commercial raw products already revealed the presence of other types of CARs (detected by FTIR) and of unknown LMW derivatives (detected by DRI signal during the HPSEC-MALS analyses). An UHPLC-MS analysis was first performed on a λ-CO from each supplier (see selected candidates, Appendix A, Table A3) using an ion-coupled reversed-phase C18 column (heptyl ammonium formate- RPIR) coupled to an ESI q-TOF MS in negative polarity. Qualitative differences were detected between the ionized species on the chromatograms (Figure 4). Due to the complexity of the formulations, enhanced by the sensibility of the analysis to detect even traces of exogenous contaminations, special attention was paid to typical markers likely to be found in the composition (galactopyranose sequences, anhydro-bridge indicating different CAR types, and altered products due to the H_2_O_2_ reactivity, Supplementary Materials, Table S1). By comparing up to 35 markers, the typical λ-type sulfated-based galactopyranose sequences, including Gal(OSO_3_^−^)-Gal, Gal(OSO_3_^−^)-Gal-Gal(OSO_3_^−^), Gal(OSO_3_^−^)-Gal-Gal-(OSO_3_^−^), or Gal(OSO_3_^−^)-Gal (Supplementary Materials, Table S1), were found in all the selected λ-COs. These markers were substituted with sulfate groups, confirming that the depolymerization reaction did not cause a complete desulfation for these OSs. Surprisingly, some sequences with higher sulfate substitution than expected were identified (a Gal(OSO_3_^−^)-Gal-Gal-(OSO_3_^−^)4 marker was found). The galactopyranose sequences detected were limited to DP3, but it is difficult to know if it is related to the depolymerization reaction, the MS analysis condition (lack of ionization of species of higher DP), or the fragmentation in the source because of highly unstable moieties. The presence of other types of CARs, with the detection of markers comprising anhydrobridge—for instance, Gal(OSO_3_^−^)-Gal-AnGal and Gal(OSO_3_^−^)-Gal-AnGal-(OSO_3_^−^)—was confirmed. In all the formulations, the presence of more complex, strongly oxidized, or modified species, such as lactone chemical groups or galactonic acid or destructured polyols was also detected (Supplementary Materials, Table S1). Such by-products are often present when the H_2_O_2_ depolymerization method is used, due to the high reactivity of the reagent that modifies the sugar backbones [60]. Furthermore, this confirms the previous results of the quantification of the reducing sugar ends that suggested structural changes at the reducing ends.

**Figure 5:**
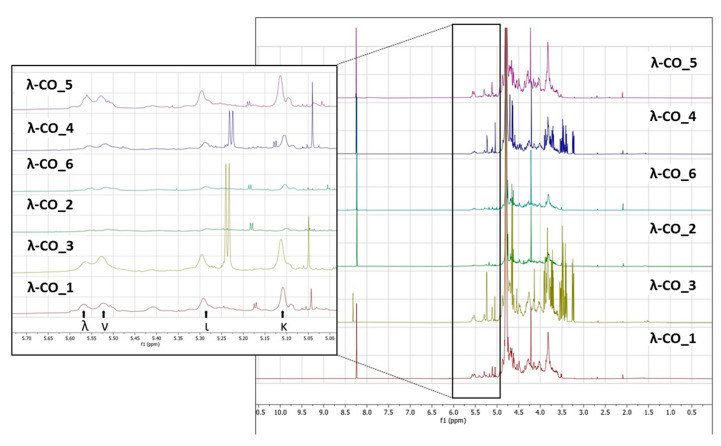
^1^H NMR spectra (500 MHz) in D_2_O of a selected λ-CO from each supplier.

To support these data, a ^1^H NMR (500 MHz) analysis in D_2_O of a selected λ-CO from each supplier was performed. Interestingly, notable differences were detected in the different spectra (Figure 5). In particular, it was clearly observed in the region at 3.6–3.2 ppm that the λ-CO from supplier_3 and supplier_4 contained glucose (Triplet at 3.2 ppm attributed to the H_2_ of glucose). The use of this additive is common in manufacturing processes to control the final viscosity of the mixture [58]. The ratio of glucose/CARs was estimated by semi-quantification for both suppliers, and these formulations were estimated to contain about 25% of glucose. Again, this observation reinforced several results presented before. Indeed, this can explain why these two commercial λ-CARs have a lower DS compared with the others, and the particularly strong intensities of the additional peaks that correspond to LMW species, detected by DRI on the chromatograms. It also confirmed the results obtained with the quantification of reducing ends that showed an unexpected amount of reducing sugars at the beginning of the λ-CARs depolymerization from these two suppliers. Moreover, it seems that the presence of glucose can reduce the kinetics of the depolymerization at the early stages of the reaction. This could be explained by a possible interaction with the depolymerizing agent. To confirm this, we performed a hydrolysis on λ-CARs from a supplier where we did not detect glucose, with and without the artificial addition of glucose in the solution. However, in this case, a slowdown of the reaction’s kinetics was not obtained (data not shown). A more detailed analysis revealed that all formulations contained the other various usual CAR types in addition to the λ-type, confirming the results of the FTIR analysis. The region 5.7–5.0 ppm is crucial to determine the proportion of each type of CARs by ^1^H NMR (inset in Figure 5) because it corresponds to the anomeric Hα of the D unit with different shielding, depending on the sulfate substitution and anydro-bridge of each type of CARs [58,61,62]. The lowest peaks at 5.55 and 5.52 ppm correspond to λ and ν CARs, respectively, due to the sulfate groups on C_2_ and C_6_. The peaks at 5.29 and 5.11 ppm correspond to the ι and κ CARs, respectively, which are less deshielded due to the anhydro-bridge on C_3_-C_6_ and, for κ, due to the presence of the OH group on C_2_. A semi-quantification was then performed to determine the proportion of each type of CARs (Table 5). The mixtures from supplier_4 and supplier_5 contained the lowest proportion of λ-type with only ~17%, while the mixture from supplier_6 contained the highest proportion with 23%. Moreover, in the products from supplier_2 and supplier_6, the ν-type, which is very close to the λ structure, was the most represented, whereas the κ type predominated in the four other products. It must be noted that the analysis does not differentiate if κ/ι types correspond to hybrid or single species. 

**Figure 6:**
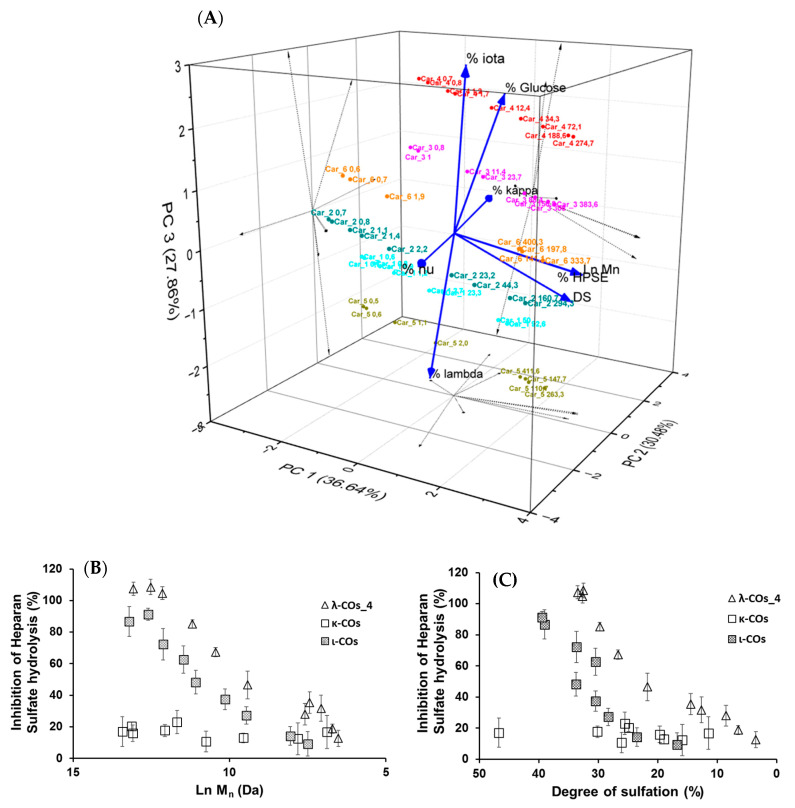
(**A**) Principal component analysis of the multivariate physicochemical features of the λ-COs and their inhibition of the HPSE activity. Impact of the (**B**) M_n_ and (**C**) DS on the anti-HPSE activity of κ and ι-COs compared with those of λ-COs_4, measured at 2.5 µg·mL^−1^.

Based on these results, it was hypothesized that the presence of glucose and the proportion of the different types of CARs in the different formulations could explain the variations in HPSE activity inhibition between the products from different suppliers. In an attempt to complete the picture, a multivariate principal component analysis of all parameters was performed. Results indicated that three principal components with close Eigen-values were needed to capture 95% of the variance: PC1 (2.929, 36.6%), PC2 (2.439, 30.5%), and PC3 (2.228, 27.9%) (Figure 6A). As expected, the M_n_ and DS were identified as the main drivers of HPSE activity inhibition with a very good correlation, and their variance was almost entirely integrated into PC1. Second, the λ-COs could be classified into groups corresponding to their initial supplier. The presence of glucose drew logically the λ-COs from supplier_4 and supplier_3 groups in the PC2/PC3 axis, which were further subdivided according to the proportion of ι (variance mainly in PC3) and κ (variance mainly in PC2) types, respectively. The proportion of ν-type appeared to be an important input to distinguish the λ-COs from supplier _6 and supplier_2 groups in the PC2 axis, while the variance in the proportion of λ-type was mainly included in PC3 to differentiate λ-COs from supplier_1 and supplier_5 groups. It is interesting to note these two opposites pairs of correlation, i.e., % of κ-type vs% of ν-type and% of λ-type vs% of ι-type. Indeed, strong Pearson correlations were observed (Appendix A, Table A4), i.e., −0.979 and −0.913 (*p*-values < 1 × 10^−5^). This is consistent with the fact that mu (μ) and ν-types are the precursors of κ/ι types and are converted into these types after alkaline treatment. On the other hand, the κ/ι types are also inversely correlated to the λ type, depending on the proportion of tetrasporophyte/gametophyte forms (from which they are extracted) collected to prepare the crude samples. Finally, it was also observed that glucose was added to the two samples that contained the highest level of κ/ι gelling CARs compared with non-gelling CARs (μ, ν, and λ). In fact, these additives are precisely added to balance and standardize the viscosity of the final product. 

Subsequently, the impact of variation in the composition of CAR types from different suppliers on the anti-HPSE activity was considered. First, the λ-type was the only one that weakly correlated with both axes that contained the minor variance in HPSE activity, i.e., PC2 and PC3 (Figure 6A and Appendix A, Table A4). However, these contributions were masked in this analysis by the predominant input of the M_n_ and DS. Therefore, we tried to replace these parameters with their respective coefficients (shown in Table 3) that described the general trend for the selected λ-CO from each supplier, and then to assess a possible Pearson correlation. No conclusive results were found, although a trend indicated that higher Ln (M_n_) coefficients were associated with a higher proportion of ν-type and a low proportion of κ-type (data not shown). 

We still assumed that the proportion of the different types of CARs could represent a minor parameter with respect to the ability to inhibit the HPSE activity of the different formulations. Therefore, κ-CO and ι-CO were produced in the same way as λ-Cos, and their anti-HPSE activity was monitored as a function of their M_n_ and DS (Figure 6B,C). Surprisingly, the κ-CO did not show any inhibition of the HPSE activity, regardless of their M_n_ or DS, even those produced after a short depolymerization time, as previously shown with native κ-CAR [63]. On the other hand, the ι-CO followed a similar trend to that of the λ-COs (decrease along with M_n_ and DS) but with an overall lower anti-HPSE activity. To confirm this result, IC_50_ was calculated for a candidate κ-CO (M_n_ 2.8 kDa, DS 15.9 ± 0.1%) and a ι-CO (M_n_ 3.2 kDa, DS 23.0 ± 1%). The values found were 206 ± 51 µg·mL^−1^ and 126 ± 25 µg·mL^−1^ (Appendix A, Figure A6), confirming the above-mentioned behaviors for these types of CARs. Overall, we could state that the proportion of true λ-CAR-diads in the formulations will clearly determine the effectiveness of the inhibition of the HPSE activity, but through a complex balance with other parameters, such as the (relatively) favorable presence of the ι-type, but also an unnecessary contribution from the proportion of κ-type included in the mixture. This may be because λ- and ι -CARs or their LMW derivatives present higher DS than κ-CARs-based products, which is a parameter widely associated with better anti-HPSE activity for OS-based inhibitors [41]. Two previous articles already compared the efficiency of different CAR types in inhibiting HPSE [46,47]. Both provided preliminary evidence that λ-CARs or their LMW derivatives show higher efficiency than the ones of κ-type, which was consistent with our findings.

### 2.5. Docking Studies

**Figure 7:**
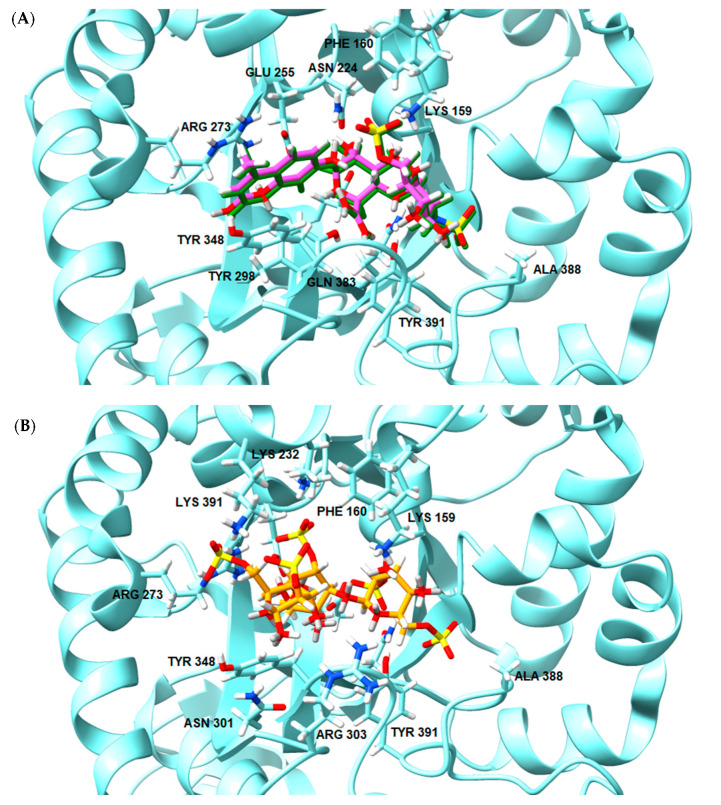
(**A**) Overlay of the ligand in the crystallographic pose (in green) and after redocking (in pink). (**B**) Most stable conformation of the best ligand λ-2 (orange) in the binding site of 6ZDM.

To go further into different capacities of the CARs to inhibit HPSE, a preliminary molecular docking approach was implemented. As a first set, the ligands used were the typical trisaccharide sequences that can be found in the λ-, κ- and ι- carrageenan characterized as Kappa 1 and 2 (κ-1 and κ-2), Iota 1 and 2 (ι-1 and ι-2) and Lambda 1 and 2 (λ-1 and λ-2) (Table 6). The crystallographic structure chosen for the calculations was the PDB code 6ZDM (Resolution = 1.71 Å), which corresponds to human heparanase complexed with a heparan sulfate disaccharide [64,65]. To the best of our knowledge, this is the first and unique structure related to containing a saccharide sequence complexed in the enzyme, so the analysis was performed on this specific cleft, assuming the CARs could equally preferentially interact there. The RMSD value obtained for redocking was 0.407, and the co-crystallographic ligand obtained a score value of 101.99 (Figure 7A). The scores and 2D structures for each molecule are presented in Table 6.

It can be seen that the most stable complex corresponds to the compound λ-2, followed by ι-1 and λ-1, which is consistent with the in vitro data discussed, where the proportion of the λ type found in the mixture contributed significantly to the overall inhibition of HPSE activity. The κ-type presented a worse performance in affinity evaluation, which is also in line with the previous results obtained for HPSE inhibition. Figure 7B shows the interaction profile for λ-2, where several amino acids equal to those shown in the crystallographic saccharide binding region can be observed. These results, taken relatively, confirm a greater efficiency of the λ class in HPSE enzyme inhibition in this case. This assumption should be qualified by the fact that for oligosaccharides, it is difficult to perform calculations for DP higher than three or four, given the complexity of possible interactions. It is also important to note that other sites or types of interactions may be at work, which is usual for this kind of complex macromolecules [40].

### 2.6. Anti-Migratory Activity on Huh7 Hepatocarcinoma Cells

**Figure 8:**
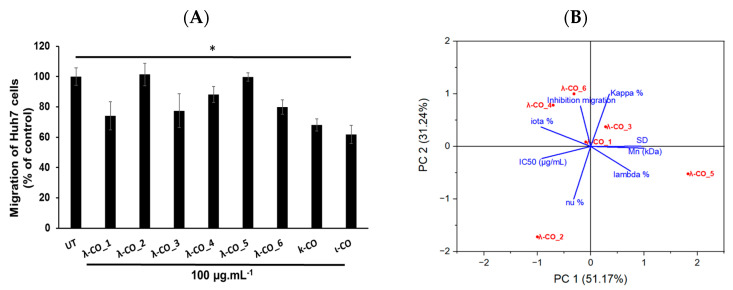
(**A**) Effect of a selected λ-CO from each supplier, κ-CO and ι-CO (at 100 μg·mL^−1^) on the migratory activity of Huh7 cells. The data are representative as the mean (±SEM of the errors mean) from three independent experiments, with at least four replicates, * *p* < 0.05, and one-way Anova Bonferroni test with mean ± SD of the three independent experiments. (**B**) Principal component analysis of the multivariate physicochemical features of the selected λ-COs, and their anti-HPSE and anti-migratory activities.

In regard to preliminary results obtained on the ability of λ-COs to reduce the migration of breast [43] and liver cancer cells (data not published), we chose to check this specific hallmark of cancer cells once again. For this experiment, the Huh7 hepatocarcinoma cell model has been chosen since for this model, it has been well described that overexpressed HPSE, among other biomolecular actors, participates in their invasiveness behaviors. The aim was to know whether the anti-migratory effects of λ-COs were correlated to their anti-HPSE activities or other interacting partners, such as chemokines, and to know whether the effects also differed according to the physicochemical parameters or to the different compositions depending on the suppliers studied above [66,67]. In addition, this allowed to investigate if the anti-migratory effects of our λ-COs were cell model dependent compared with the results previously published on MDA-MB-231 breast cancer line [43,44]. The anti-migratory effect of the selected λ-COs candidates based on the M_n_, DS and anti-HPSE IC_50_ values previously reported as suitable for in vivo anticancer applications was tested [43,44], and for comparison and discussion purposes, κ-CO and ι-CO were also tested (selected candidates, see Appendix A, Table A3). The results showed differences in the effect of the λ-COs on Huh7 cell migration depending on their origin. The λ-CO from supplier_1 had the greatest inhibitory effect of 26%, followed by λ-CO from supplier_3 with 22%, the λ-CO from supplier_6 with 20%, and finally, the λ-CO from supplier_4 with 12%; whereas, no effect was observed when the cells were treated with the λ-COs from supplier_2 and supplier_5 (Figure 8A). 

Again, a principal component analysis to try to discriminate the parameters involved in the effective inhibition of Huh7 cells migration was performed (Figure 8B). The two principal components accounted for 82% of the variance of the system. Interestingly, while the IC_50_ of HPSE inhibition was related to an anti-migratory effect (inversely related on the graph as a small IC_50_ is equivalent to strong inhibition), the presence of κ and ι types in the formulations also appeared to be an important triggering factor (Figure 8B). Indeed, Pearson correlation between the IC_50_ and the cell-migration inhibition showed only a weak relationship (data not shown). Furthermore, the strongest effects were obtained with κ-CO and ι-CO, with 32 and 38% of inhibition, respectively, confirming the previous observations. In contrast, the λ and ν types did not appear to correlate with cell migration inhibition. However, the M_n_, DS, and proportion of λ-type correlated consistently with the IC_50_ in PC1 (this correlation was demonstrated above), but they did not contribute to PC2, where most of the of variance regarding the migration inhibition was observed. This might be in line with other experiments made with λ-COs from supplier_1, which demonstrated that the M_n_ and DS did not directly correlate with the anti-migratory activity on in vitro cell models [44]. In addition, several studies have already shown that these COs have multiple activities and could inhibit cell migration by mechanisms other than the inhibition of the HPSE activity [30,50]. Based on all the results, we could assume that the anti-HPSE activity of the formulations participates in the anti-migratory effect observed in cell models, although other important mechanisms of action are certainly involved, especially those related to the κ species.

## 3. Materials and Methods

### 3.1. Native λ-Carrageenan (λ-CAR) Samples

Crude native λ-CARs were purchased from five different suppliers (Santa Cruz Biotechnology, Cargill, Merck Sigma-Aldrich, BioSynth, and FMC). They were named randomly in this article as followed (note: the order does not correspond to the one of the supplier’s list provided above): λ-CAR from supplier_1 (λ-CAR_1), λ–CAR from supplier_2 (λ-CAR_2), λ-CAR from supplier_3 (λ-CAR_3), λ-CAR from supplier_4 (λ-CAR_4), λ-CAR from supplier_5 (λ-CAR_5), and λ-CAR from supplier_6 (λ-CAR_6). 

### 3.2. Generation of λ-COs from Native λ-CARs

Native λ-CARs from each supplier were depolymerized using a previously published radical H_2_O_2_-based hydrolysis method [44]. Briefly, native λ-CARs were dissolved in Milli-Q water at a concentration of 5 mg·mL^−1^, and then the solutions were purged with argon. Then, H_2_O_2_ 30% (Sigma-Aldrich, Saint Louis, MO, USA) at a weight/weight ratio of 1.5 was added and the reaction mixture was immediately incubated at 60 °C. Fractions (10 mL) were collected at different times and were dry-frozen prior to analysis.

### 3.3. High-Pressure Size-Exclusion Chromatography with Multi-Angle Light Scattering (HPSEC-MALS) Analyses on Native λ-CARs and Selected λ-COs

**Figure A5:**
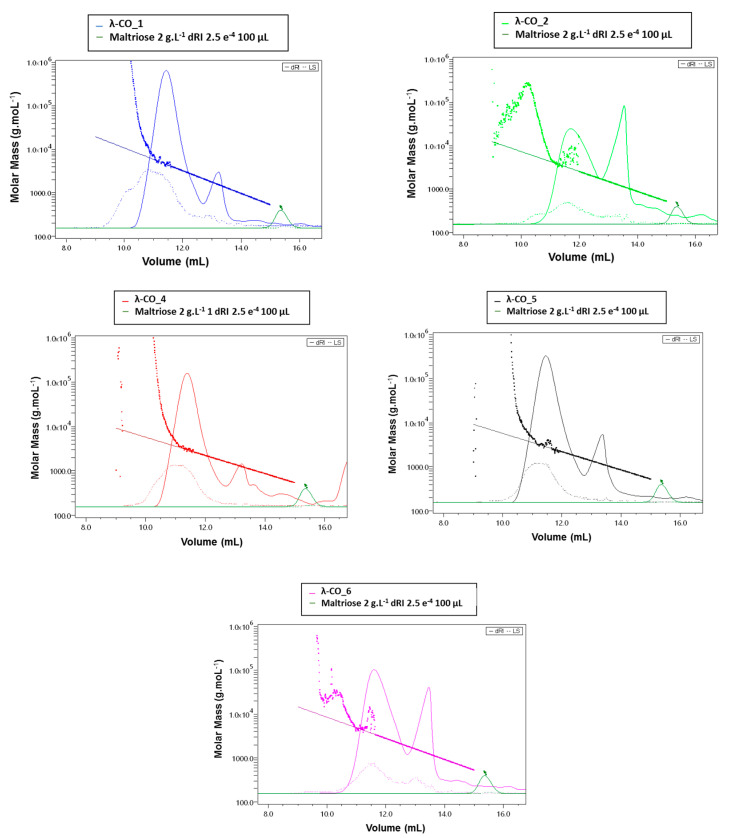
Absolute weight calculation by HPSEC-MALs analysis of a selected λ-CO from 5 suppliers.

**Figure A6:**
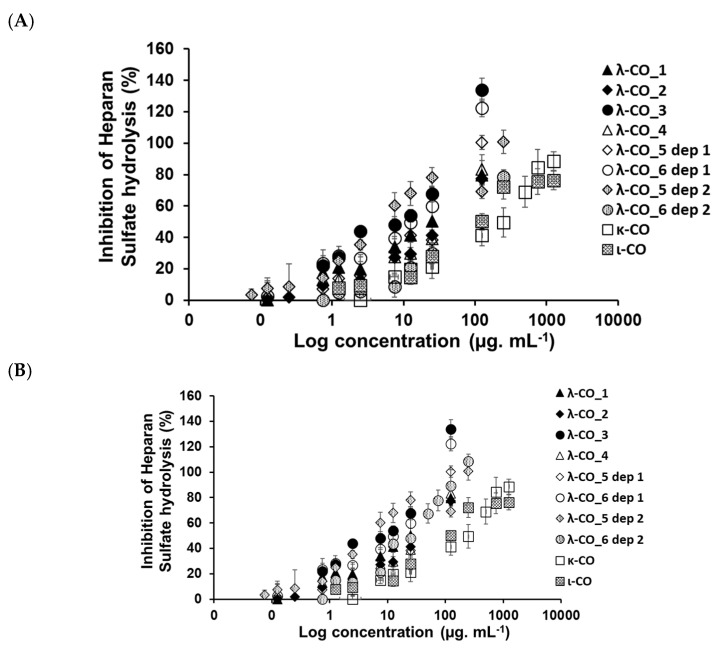
Half-maximal concentration for HPSE inhibition of (**A**) a selected λ-CO from each supplier, selected κ-CO and ι-CO, and (**B**) the native λ-CAR from each supplier.

HPSEC-MALS was performed on native λ-CARs and their corresponding selected λ-COs (Appendix A, Figure A1 and Figure A5, and Table A2) to estimate macromolecular magnitudes (the number-averaged molecular mass (M_n_), the weight-averaged molecular mass (M_w_), and the polydispersity index (Ð = M_w_/M_n_), in diluted mixtures), according to a previously published procedure [68]. The system was equipped with two detectors in line: (i) a multi-angle light scattering (MALS) filled with a He–Ne laser (λ = 690 nm) and a K5 cell (50 µL) (HELEOS II Wyatt Technology Corp, Santa Barbara, CA, USA); (ii) a differential refractive index (DRI) (RID10 A Shimadzu, Kyoto, Japan). The solvent (0.1 M LiNO_3_) was filtered through a 0.1 µm filter unit (Millipore, and degassed (DGU-20A3, Shimadzu, Kyoto, Japan). The SEC line was composed of an OHPAK SB-G guard column and three OHPAK SB 806, 804, and 802.5 (for native λ-CARs) or 802 and 802.5 (for the selected λ-COs) HQ columns (Shodex Showa Denko K.K, Tokyo, Japan) eluted in series with a LiNO_3_ solution (0.1 M) at a flow rate of 0.5 mL·min^−1^. Native λ-CARs from each supplier and their corresponding selected λ-COs were solubilized for 48 h at a concentration of 1 and 15 g·L^−1^ in 0.1 M LiNO_3_ solution, respectively, filtered (0.45 µm), and then injected through a 100 µL full loop (SIL-20A, Shimadzu, Kyoto, Japan). Astra 6.1.7.16 software was used to analyze the data using a DRI dn/dc of 0.125 mL·g^−1^ [69].

### 3.4. Structural and Quantitative Analysis by HPSEC with DRI (HPSEC-DRI) of the Generated λ-COs

The structural and quantitative analyses of the generated λ-COs were performed by HPSEC using two successive analytical columns (TSK-GEL G4000PW and TSK-GEL G3000PWXL, Tosoh, Japan) coupled with an LC system from Agilent (Santa Clara, CA, USA) equipped with a DRI (Agilent, Santa Clara, CA, USA). Products were isocratically eluted with 0.1 M sodium nitrate (NaNO_3_) solution at a flow rate of 0.8 mL·min^−1^. The M_n_, M_w_, number-averaged degree of polymerization (DP (with a monomer unit of 160.5 Da)), and Ð were calculated using a previously published method [44]. The chromatograms were calibrated using a series of pullulan standards (from 1,3 kDa to 806 kDa, Polymer Standards Service GmbH, Mainz, Germany). For fractions below 10 kDa, their molar mass equivalents were calculated using heparin standards (from 1200 Da to 5750 Da, Iduron, UK).

### 3.5. Determination of the Degree of Sulfation (DS) 

The sulfate content of native λ-CARs was determined by inductively coupled plasma mass spectrometry (ICP-MS). Briefly, 250 mg of each product were dissolved in a 6:2 (*v*/*v*) 67–70% HNO_3_/34–37% HCl mixture (Fisher, trace metal quality). Acidic digestion of the samples was carried out overnight at room temperature and then in a Milestone Ehos Up microwave oven (30 min with constantly increasing temperature up to 120 °C, then 15 min at this temperature). Each sample was made up to 50 mL with ultrapure quality water. The analyses were performed with an Agilent 5800VDV ICP-AES and a Thermofisher Scientific XSeries 2 ICP-MS (Waltham, MA, USA). 

Regarding the generated λ-COs, the sulfate content was determined using a (7-aminophenothiazin-3-ylidene)-dimethylazanium chloride (Azure A, Sigma-Aldrich, Saint Louis, MO, USA) based colorimetric assay, which binds covalently to sulfated groups on the sugar backbone to form a colored complex [70]. Briefly, 20 µL of three dilutions (0.03, 0.04, 0.05 mg·mL^−1^) of each sample were added to a 96-well plate. Then, 200 µL of 10 mg. L^−1^ AzureA aqueous solution was added, and the absorbance was measured at 630 nm after 10 min of incubation. The DS was calculated from a calibration curve using absorbance values obtained from a serial dilution (0–0.03 mg·mL^−1^) of dextran sulfate standard (Sigma-Aldrich, Saint Louis, MO, USA) with a known sulfur content of 18.1%.

### 3.6. Fourier-Transform Infrared (FTIR) Spectroscopy Measurements of the Native λ-CARs

The FTIR spectra of the native λ-CAR powders were obtained using a PerkinElmer UATR Two FTIR spectrometer; 64 scans per spectrum; nominal resolution: 2 cm^−1^; scan speed: 0.2 cm·s^−1^; apodization: strong; source: MIR (8.000–30) cm^−1^; detector: MIR TGS (15.000–370) cm^−1^; the background was subtracted for each sample and a baseline correction was applied. All the spectra were recorded in the 4.000–450 cm^−1^ region, although only the 1.500–500 cm^−1^ area was used for the spectral analysis.

### 3.7. Structural Characterization by Ultra-Performance Liquid Chromatography Tandem Mass Spectrometer (UPLC-MS) and NMR Analyses of the Selected λ-COs

Analyses were performed using a UPLC system “Acquity UPLC H-class” (Waters, Milford, USA) coupled to a high-resolution mass spectrometer “XEVO G2-S QTof” equipped with an electrospray ionization (ESI) source (Waters, Manchester, UK). The UPLC system consisted of a quaternary pump (Quaternary Solvent Manager, Waters) and an autosampler (Sample Manager-FTN, Waters) equipped with a 10 µL injection loop. After dilution in an aqueous solution of 5 nM heptylammonium formate (at 1 mg·mL^−1^), 10 µL of the selected λ-COs from each supplier (see selected candidates, Appendix A, Table A3) were injected into an Acquity UPLC BEH C18 column (50 mm × 2.1 mm, 1.7 µm; Waters), then heated at 45 °C. The system was operated at 0.35 mL·min^−1^ under the following elution gradient program, involving solvent A (5 nM heptylammonium in H_2_O, 0.0238% (*v*/*v*) formic acid, pH = 4.0) and solvent B (5 Nm heptylammonium in MeOH/H_2_O (3:1, *v*/*v*), 0.0238% (*v*/*v*) formic acid, pH = 4.0) as following: 0–4 min, 10–50% B; 4–6 min, 50% B; 6–10 min, 50–80% B; 10–15 min, 80% B; 16–27 min, 100% B; 28–34 min, 10% B. The column and the autosampler were maintained at +45 °C and +10 °C, respectively. Eluted compounds were ionized in the negative polarity (ESI^−^) with the following parameters: capillary voltage 0.5 kV, source temperature 120 °C, source offset 80 V, desolvation temperature 500 °C, cone gas flow 50 L·h^−1^, and desolvation gas flow 800 L·h^−1^. The MS and MS/MS analyses were performed using either the MS^E^ or the MS/MS fast data-dependent acquisition (fastDDA) approaches (Waters, Manchester, UK) in a centroid mode with a time scan of 0.25 sec/scan. An MS^E^ experiment consists of data acquisitions in a single same run, with no collision energy in function 1, without fragmentation (low energy function), and a ramped collision energy in function 2, to generate fragment ions (high energy function). MS^E^ software algorithms then assign fragment ions spectra to their associated ion precursor peak by profiling each chromatographic peak and determining their retention time. An MS/MS fastDDA experiment consists of acquisitions in a single same run, with a selection of precursor ions when their intensity rises above 50.000 a.u./s, during 5 s or until their intensity falls below 1.000 a.u./s., and a ramped collision energy to generate fragment ions. Both MS^E^ function 2 and MS/MS fastDDA acquisitions were performed with a ramped collision energy of 15–60 eV. Leucine Enkephalin (M = 555.62 Da, 1 ng·µL^−1^) was used as a lock-mass shift correction, and the mass spectrometer was calibrated before the analyses using a 0.5 mM sodium formate solution.

For NMR, the lyophilized powders of the selected λ-COs were redissolved in a D_2_O solution at a concentration of about 20 mg·mL^−1^. NMR spectra were recorded using a Bruker AVANCE III 500 MHz spectrometer at room temperature. Experiments of ^1^H (64 scans, 10 min), ^13^C (4,096 scans, 4 h 44 min), correlation spectroscopy COSY (32 scans, 3 h), and heteronuclear ^1^H/^13^C chemical shift correlation (HMQC) (32 scans, 3 h) were performed using the Bruker’s conventional pulse programs. Spectra were analyzed with a fee-trial version of MestreNova. Chemical shifts were expressed in ppm relative to the deuterated peak solvent (4.65 ppm), and identifications were based on previous work in the field. 

### 3.8. Heparanase (HPSE) Enzyme Activity Assay

The inhibition of HPSE activity and the half-maximal inhibitory concentrations (IC_50_) were assessed using the HPSE assay toolbox (Cisbio Assay, Codolet, France) and human recombinant HPSE-1 (R&D Systems, Minneapolis, MN, USA), as previously described [44], based on the principle of fluorescence resonance energy transfer (FRET). Briefly, 15 µL of the λ-CAR, λ-CO, κ-CO, or ι-CO powder dissolved in Milli-Q water (at 2.5 µg·mL^−1^ for monitoring assay and at 0.025–1250 µg·mL^−1^ range for IC_50_ calculation) were added into the wells, followed by 15 μL of the HPSE-1 solution (100 ng·mL^–1^). The enzyme reaction was initiated after 10 min preincubation at 37 °C, by adding 30 μL of a Biotin-HS-Eu(K) solution and the plate was incubated at 37 °C for 15 min. Then, 20 μL of a streptavidin-XL665 solution was added for the detection step. The fluorescence was measured after 5 min at λem1 = 620 nm and λem2 = 665 nm, after 60 μs of excitation at λex = 337 nm. The Delta F (%) was calculated as previously described [44].

### 3.9. Docking Studies

Molecular docking calculations were performed using GOLD 2022.3.0 package [71]. This software uses a genetic algorithm that exploits the full range of conformational flexibility of the ligand, with partial flexibility of the protein [71]. The score function chosen was the ChemPLP, which runs well in both pose prediction and virtual screening, as noted in comparative studies with other GOLD score functions [72]. Higher values of the calculated score denote the most stable poses for a ligand. The calculations were performed considering the coordinates centered at the co-crystallographic ligand, with X = −20.63, Y = 18.087, and Z = 58.802, respectively. The crystallographic structure chosen for the calculations was the PDB code 6ZDM (Resolution = 1.71 Å), downloaded from the Protein Data Bank (www.rcsb.org), which corresponds to human heparanase complexed with a heparan sulfate disaccharide [65,73]. The removal of the co-crystallographic ligands and the addition of hydrogens was performed using Chimera software [74]. All the ligands were drawn on Marvin Sketch 17.21.0, Chemaxon (https://www.chemaxon.com, accessed on 10 April 2023), and had their protonation state set to pH 5.5 using the major species plugin of the same software. In the case of the protein, the pH was adjusted in APBS online platform (Adaptive Poisson-Boltzmann Solve) [75]. After being drawn in 2D and converted to 3D, all molecules were optimized with the RM1 semi-empirical method using the MOPAC software, J. J. P. Stewart, Stewart Computational Chemistry: Colorado Springs, CO, USA (http://OpenMOPAC.net, accessed on 10 April 2023).

### 3.10. Cell Migration Assays on Huh7 Hepatocarcinoma Cells

Cell migration was assessed on 2.5 × 10^5^ Huh7 cells with BioCoat cell migration chambers (Corning, Amsterdam, The Netherlands). Briefly, inserts of BioCoat cell migration chamber were coated with fibronectin (100 μg·mL^−1^; Corning, Amsterdam, The Netherlands). Then, 2.5 × 10^5^ Huh7 cells were resuspended in basal DMEM low-glucose media (Invitrogen, Fisher Scientific Illkirch, France). Cells were added in the upper chamber, and complete media with 10% of Fœtal Calf Serum for “Untreated Cells” or complete media with 100 µg·mL^−1^ of selected λ-COs or controls (Appendix A, Table A3) were added in the lower chamber. After 24 h, cells that had migrated through the porous membrane were stained for 30 min with 1% crystal violet (Sigma-Aldrich, Saint-Quentin Fallavier, France) and counted manually by two different observers who performed blindly acquired data.

### 3.11. Statistical Analyses

One-factor linear regression was performed by Excel software. Pearson model of covariance and correlation, a two-factors multiple linear regression method (least square method), and a principal component analysis were performed using a free-trial version of Minitab or Origin. Statistical significance of the effects of the sugar candidates on the cell migration was assessed using a one-way Anova Bonferonni test on the GraphPad Prism software with *p*-values ≤ 0.05.

## 4. Conclusions

Many studies already consider λ-COs as very promising candidates for various biomedical applications. However, similar to polysaccharides extracted from natural resources, the physicochemical structure of native parent λ-CAR could depend on the algal sources, life stage, the environment in which they grow, or the industrial extraction method used. More importantly, a key factor often overlooked in this field is that though sold commercial products are based on a functional definition of viscosity, they are actually a mixture of different molecular species, especially other types of CARs. In this study, we explored several issues regarding the λ-CAR starting raw material used to produce bioactive λ-COs with an anti-HPSE activity through an H_2_O_2_-based depolymerization reaction and their effect on cancer cell migration. First, we found strong disparities between the λ-CAR of six commercial suppliers with respect to their physicochemical features (M_n_, DS, and Ð), but also with respect to the composition of the mixtures. As expected, we found varying proportions of κ-, ι-, and ν-types, and other types of CARs in the same or even higher proportions than the λ-type. In addition, an important amount of glucose was detected in two out of the six products. This additive is common in products intended for agro-industrial or cosmetic applications. However, when biological activities are analyzed, this can bias the obtained results. In biomedical studies and the production of bioactive λ-COs, all these species will be present in the LMW formulations and it could be difficult to purify them. Therefore, a first recommendation is to use a λ-CAR self-extracted and self-purified from tetrasporophyte algae form or to perform a pre-purification step before depolymerization if purchased from a commercial supplier. Furthermore, the results already described in previous studies that have used a commercial product should be interpreted with caution as the effects could also be due to other types of CARs. When we applied H_2_O_2_-based depolymerization, desulfation occurred along with chain length reduction, which is characteristic of these radical-based methods. This leads to the generation of OS formulas that could be considered as λ-CAR derivatives different from the initial structure of the native product. In fact, the depolymerization modified the sugar backbones by creating new chemical groups, as shown by the MS analysis. It is difficult to determine the impact of these changes, which could result in additional heterogeneity but also in new or enhanced bioactivities because of these original groups. Still, we showed that it is possible to obtain nearly similar formulations in terms of M_n_, M_w_, and DS (corresponding to the means of all species present in the mixture) despite initial differences between the suppliers, provided that the depolymerization time is adjusted and that close real-time monitoring is performed. This real-time monitoring is also important to ensure reproducibility of the mean M_n_, M_w_, and DS of the λ-COs produced, which is not possible with a similar reaction time in our experimental conditions but should be possible in advanced bioreactors and working with a slightly lower concentration of λ-CAR. Here, we investigated the production of λ-COs for their ability to inhibit the enzymatic activity of HPSE, a pharmaceutical property of the λ-CAR that is of increasing interest in medical research. As expected, and already widely reported, the inhibition of HPSE activity is strongly correlated with decreases in mean M_n_ and DS of the formulations. Nonetheless, it was difficult to discern if one of the parameters was more important than the other for the λ-COs ability to inhibit HPSE. Further research should be conducted, for instance by applying a non-desulfating depolymerization method or by performing gradual re-sulfation on selected λ-COs by chemical methods. We also showed that while the proportion of λ-type found in the mixture contributed significantly to the overall inhibition of the HPSE activity by the formulations, the ι-COs present in significant proportion also participated to a lesser extent. On the other hand, specifically with respect to the inhibition of the HPSE activity, the proportion of κ-type would represent a significant loss. The inhibition of the HPSE activity by the μ and ν types of CARs should be also examined in future studies to get a complete picture, as they could also represent significant contributors to the final HPSE inhibition. Finally, the evaluation of the effects of λ-COs on Huh7 cell migration showed that these effects did not fully correlate with their anti-HPSE activities. These results also confirmed that while HPSE targeting is very promising, it is only suitable in specific well-determined biological contexts in order to obtain adequate and controllable effects. In general, the biomedical use of OSs is already characterized by multiple potential synergic activities and by the difficulty of determining their various molecular mechanisms due to their structure complexity. In this direction, further research should be conducted to decipher the mechanism of these candidates in the biological background of Huh7. For λ-COs produced from native λ-type products, an additional challenge is that they actually correspond to a mixture of different macromolecular species, which multiplies the possibilities and hypotheses to explain the biological need. Therefore, a commercial extraction only from tetrasporophyte algae or a new industrial pre-purification process should represent a major step in the field and toward further medical applications. 

## Figures and Tables

**Table 1:** Molar masses (M_n_ and M_w_), number-averaged degree of polymerization (DP), and polydispersity index (Ð) from the HPSEC-MALS analyses, and degree of sulfation (DS) of commercial native λ-CARs. M_n_ and M_w_ values are given with an error of 5%.

Native λ-CAR Supplier	M_n_ (kDa)	M_w_ (kDa)	DP	Ð	DS% (*w*/*w*)
λ-CAR_1	215	762	871	3.5	26.0 ± 1.2
λ-CAR_2	500	850	1900	1.7	27.0 ± 1.2
λ-CAR_3	597	964	2607	1.6	21.4 ± 0.2
λ-CAR_4	279	711	1156	2.5	21.9 ± 0.9
λ-CAR_5	392	773	1465	2.0	28.5 ± 0.7
λ-CAR_6	289	600	1080	2.1	28.9 ± 0.2

**Table 2:** Molar masses (M_n_ and M_w_), number-averaged degree of polymerization (DP), and polydispersity index (Ð) calculated with HPSEC-DRI analyses (using pullulan or heparin (*) standards), and degree of sulfation (DS) of all the λ-COs generated from the six commercial native λ-CARs. dep (1): first depolymerization. dep (2): second depolymerization.

λ-COs	Time (h)	M_n_ (kDa)	M_w_ (kDa)	DP	Ð	DS% (*w*/*w*)
**λ**-COs_1	2	92.6	142.7	337	1.5	41.5 ± 0.4
	4	50.0	74.5	184	1.5	41.0 ± 2.9
	8	23.3	31.6	114	1.4	21.0 ± 2.2
	12	2.7 *	4.7 *	12 *	1.8	27.5 ± 0.9
	dep (1) 24	1.2 *	1.8 *	6 *	1.5 *	14.0 ± 1.5
	dep (2) 24	2.3 *	4.0 *	11 *	1.8 *	23.8 ± 0.7
	36	0.9 *	1.2 *	5 *	1.5 *	10.0 ± 1.4
	48	0.8 *	1.1 *	4 *	1.4 *	8.0 ± 1.1
	72	0.7 *	0.9 *	4 *	1.3 *	2.7 ± 0.4
	96	0.6 *	0.8 *	4 *	1.3 *	1.8 ± 0.4
**λ**-COs_2	2	294.3	390.9	1069	1.3	42.0 ± 1.4
	4	160.7	236.2	579	1.5	42.2 ± 0.8
	8	44.3	65.6	167	1.5	40.0 ± 1.3
	12	23.2	31.7	92	1.4	36.0 ± 1.8
	24	2.0 *	3.7 *	10 *	1.7 *	25.0 ± 1.3
	36	1.4 *	2.4 *	7 *	1.8 *	16.8 ± 0.8
	dep (1) 48	1.1 *	1.7 *	6 *	1.6 *	13.0 ± 1.2
	dep (2) 48	1.1 *	1.8 *	6 *	1.6 *	9.8 ± 0.6
	72	0.8 *	1.2 *	5 *	1.4 *	8.0 ± 1.0
	96	0.7 *	1.0 *	4 *	1.4 *	5.5 ± 0.6
**λ**-COs_3	2	383.6	475.6	1570	1.2	34.0 ± 1.1
	4	308.0	410.5	1290	1.3	32.8 ± 0.5
	8	156.8	233.0	664	1.5	32.1 ± 0.8
	12	88.4	133.0	382	1.5	31.0 ± 1.2
	24	23.7	30.6	114	1.3	23.0 ± 2.3
	36	11.4	16.2	56	1.4	22.0 ± 2.3
	dep (1) 48	1.8 *	3.1 *	9 *	1.7 *	15.9 ± 0.7
	dep (2) 48	0.7 *	1.1 *	4 *	1.5 *	7.8 ± 0.3
	72	1.0 *	1.6 *	6 *	1.6 *	9.2 ± 0.6
	96	0.8 *	1.1 *	4 *	1.4 *	7.0 ± 1.5
**λ**-COs_4	2	274.7	368.9	1156	1.3	32.0 ± 1.2
	4	188.6	271.6	792	1.4	32.6 ± 0.3
	8	72.1	110.0	316	1.5	29.7 ± 0.4
	12	34.3	49.7	157	1.5	26.7 ± 0.1
	24	12.4	17.5	60	1.4	21.7 ± 0.8
	36	1.7 *	2.9 *	9 *	1.7 *	14.0 ± 1.1
	dep (1) 48	1.2 *	1.9 *	6 *	1.6 *	12.6 ± 0.5
	dep (2) 48	2.0 *	4.0 *	11 *	2.0 *	8.5 ± 0.4
	72	0.8 *	1.2 *	5 *	1.5 *	6.5 ± 0.5
	96	0.7 *	0.9 *	4 *	1.3 *	3.5 ± 0.5
**λ**-COs_5	2	411.6	492.8	1615	1.2	37.0 ± 1.3
	4	263.3	344.9	933	1.3	43.0 ± 2.9
	8	147.7	225.5	582	1.5	37.0 ± 1.8
	12	110.7	166.8	399	1.5	42.2 ± 0.7
	24	2.0 *	3.8 *	9 *	1.9 *	27.0 ± 1.3
	dep (1) 36	1.1 *	1.6 *	6 *	1.5 *	12.0 ± 1.1
	dep (2) 36	2.5 *	4.5 *	12 *	1.8 *	24.1 ± 0.9
	48	0.9 *	1.3 *	5 *	1.5 *	6.0 ± 1.7
	72	0.6 *	0.8 *	4 *	1.3 *	2.0 ± 1.6
	96	0.5 *	0.7 *	3 *	1.2 *	0.5 ± 0.9
**λ**-COs_6	2	400.3	496.8	1610	1.2	35.0 ± 2.6
	4	333.7	446.4	1170	1.3	44.0 ± 2.5
	8	197.8	279.4	792	1.4	36.0 ± 1.8
	12	141.1	223.9	537	1.6	39.0 ± 6.3
	24	14.1	20.4	52	1.4	41.0 ± 6.1
	dep (1) 36	1.9 *	3.5 *	10 *	1.9 *	15.9 ± 0.9
	dep (2) 36	1.4 *	2.2 *	7 *	1.6 *	18.0 ± 1.8
	48	1.2 *	1.8 *	7 *	1.6 *	10.0 ± 1.8
	72	0.7 *	1.1 *	5 *	1.4 *	5.0 ± 2.7
	96	0.6 *	0.8 *	4 *	1.3 *	2.3 ± 0.3

**Table 3:** Results of the regression between HPSE inhibition with Ln (M_n_) and DS (degree of sulfation) taken independently, and results of the multiple linear regression considering the two variables at the same time.

	Independent Single Analysis	Multiple Parametric Analysis		
	Coefficients of the Slope	Coefficients in the Equation %HPSE = a × Ln (Mn) + b × DS	*R* Squared-Variance of the System	*p*-Value
	% HPSE Inhibition vs. Ln (Mn), *r*^2^	a Ln (Mn)		
	% HPSE Inhibition vs. DS, *r*^2^	b DS		
Whole Library	15.84 (0.92)	10.04 ± 1.26	97.6%	<1 × 10^−5^
	2.59 (0.82)	1.189 ± 0.162		<1 × 10^−5^
λ-COs_1	13.85 (0.94)	7.23 ± 1.03	99.5%	2.0 × 10^−4^
	1.82 (0.96)	0.92 ± 0.099		3.6 × 10^−5^
λ-COs_2	18.49 (0.99)	14.38 ± 1.58	99.5%	3.9 × 10^−5^
	2.82 (0.91)	0.740 ± 0.165		2.8 × 10^−3^
λ-COs_3	12.75 (0.95)	6.55 ± 3.56	98.7%	0.116 *ns*
	2.83 (0.91)	1.526 ± 0.554		0.033
λ-COs_4	14.82 (0.97)	5.61 ± 2.91	98.7%	0.095 *ns*
	3.14 (0.93)	2.131 ± 0.423		1.5 × 10^−3^
λ-COs_5	16.66 (0.90)	6.52 ± 3.11	98.5%	0.081 *ns*
	2.83 (0.94)	1.804 ± 0.393		3.7 × 10^−3^
λ-COs_6	16.34 (0.96)	6.44 ± 2.71	99.5%	0.063 *ns*
	2.86 (0.97)	1.831 ± 0.379		4.7 × 10^−3^

**Table 4:** Half-maximal inhibitory concentrations (IC_50_) to inhibit the HPSE activity of a selected λ-CO from each supplier.

Selected λ-COs	M_n_ (kDa)	DS	IC_50_ (µg·mL^−1^)	IC_50_ (µg·mL^−1^) of the Corresponding Native λ-CAR
λ-CO_1	1.2	14.0 ± 1.5	24.0 ± 7.0	0.7 ± 0.1
λ-CO_2	1.1	13.0 ± 1.2	35.0 ± 9.0	0.4 ± 0.1
λ-CO_3	1.8	16.0 ± 0.7	08.0 ± 2.0	0.9 ± 0.2
λ-CO_4	1.2	12.6 ± 0.5	28.0 ± 7.0	0.7 ± 0.2
λ-CO_5 dep (1)	1.1	12.0 ± 1.1	22.0 ± 5.0	0.3 ± 0.1
λ-CO_5 dep (2)	2.5	24.1 ± 0.9	07.0 ± 2.0	
λ-CO_6 dep (1)	1.9	16.0 ± 1.0	13.0 ± 4.0	0.9 ± 0.4
λ-CO_6 dep (2)	1.4	18.0 ± 1.8	25.0 ± 5	

**Table 5:** Semi-quantitative analysis of the molar composition of the different products in terms of CAR types on a selected λ-CO from each supplier.

CAR Type	λ-CO_1	λ-CO_2	λ-CO_3	λ-CO_4	λ-CO_5	λ-CO_6
λ	22%	19%	19%	17%	23%	17%
ν	27%	35%	28%	25%	27%	33%
ι	21%	23%	22%	24%	18%	23%
κ	30%	23%	31%	34%	32%	26%

**Table 6:** ChemPLP score values and structures of trisaccharides used in docking calculations.

	κ-1	κ-2	ι-1	ι-2	λ-1	λ-2
**Score**	67.37	60.93	86.42	70.16	80.40	91.02
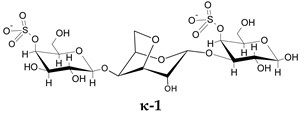				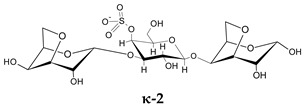		
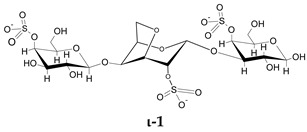				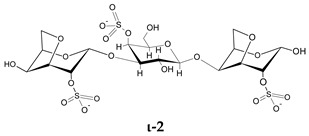		
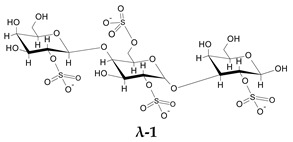				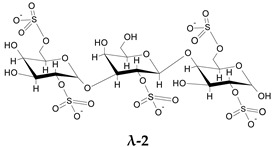		

## Data Availability

The data presented in this study are available on request from the corresponding authors.

## Supplementary Materials

The following supporting information can be downloaded at: https://www.mdpi.com/article/10.3390/md21050295/s1, Table S1: Molecular identification of the typical structures found in the formulations of a selected λ-CO from 5 suppliers.

## Appendix A

**Table A1:** Elementary analysis of native λ-CARs by ICP-MS.

Native λ-CAR Supplier	As	Cd	Fe	Mg	Mn	Na	P	Pb	S	Se	Si	Sn
λ-CAR_1	0.54	0.61	78.95	4427.16	24.80	47675.13	892.47	0.13	81173.34	<0.40	306.18	0.03
λ-CAR_2	0.55	0.34	36.61	2432.05	5.63	53037.50	417.25	0.16	84436.82	<0.40	205.1	0.02
λ-CAR_3	0.34	0.13	53.57	931.51	4.11	40002.91	2095.61	0.42	66406.10	<0.40	427.27	<0.02
λ-CAR_4	0.33	0.13	54.60	952.18	4.14	40725.85	2066.10	0.42	68283.02	<0.40	434.79	<0.02
λ-CAR_5	0.21	0.39	43.29	2075.66	11.83	43571.08	440.32	0.14	88518.93	<0.39	612.79	<0.02
λ-CAR_6	0.25	0.49	30.18	1545.34	6.25	44237.30	343.74	0.10	89823.36	< 0.38	426.57	<0.02

**Table A2:** Comparison between HPSEC-DRI and HPSEC-MALS M_n_ and M_w_ calculations of a selected λ-CO from each supplier *: M_n_ and M_w_ calculated using heparin standards.

Selected λ-CO	HPSEC-DRI		HPSEC-MALS	
	M_n_ (kDa)	M_w_ (kDa)	M_n_ (kDa)	M_w_ (kDa)
λ-CO_1	2.3 *	4.0 *	2.95 ± 0.26	3.93 ± 0.14
λ-CO_2	1.1 *	1.8 *	2.17 ± 0.91	4.26 ± 1.22
λ-CO_3	0.7 *	1.1 *	n.d	n.d
λ-CO_4	2.0 *	4.0 *	2.66 ± 0.55	3.94 ± 0.28
λ-CO_5	2.5 *	4.5 *	2.61 ± 0.56	3.34 ± 0.31
λ-CO_6	1.4 *	2.2 *	2.71 ± 1.28	4.57 ± 1.45

**Table A3:** Selection of OS candidates for each experience. *: M_n_ calculated using heparin standards.

Selected OS	IC50 HPSE	Anti-migration	NMR	MS
λ-CO_1	1.2 * kDa	1.2 * kDa	2.3 * kDa	0.9 * kDa
λ-CO_2	1.1 * kDa	1.1 * kDa	1.1 * kDa	1.4 * kDa
λ-CO_3	1.8 * kDa	1.8 * kDa	29.2 kDa	1.8 * kDa
λ-CO_4	1.2 * kDa	1.2 * kDa	2.0 * kDa	1.7 * kDa
λ-CO_5	1.1 * kDa	2.5 * kDa	2.5 * kDa	1.9 * kDa
λ-CO_6	1.9 * kDa	1.4 * kDa	1.4 * kDa	n.d
κ-CO	2.8 * kDa	2.8 * kDa	n.d	n.d
ι-CO	3.2 * kDa	3.2 * kDa	n.d	n.d

**Table A4:** Results of PCA analysis and Pearson correlations. Green represents the correlation with% of HPSE inhibition. Red represents the opposite correlation.

	PC1	PC2	PC3
Ln M_n_	0.55497	0.13043	−0.10275
DS	0.54558	0.04277	−0.18507
%HPSE	0.55948	0.11723	−0.10104
Glucose	0.05819	0.40542	0.45767
% λ-CAR	−0.18312	0.11617	−0.60582
% ν-CAR	0.13358	−0.61068	0.00732
% ι-CAR	0.13125	−0.16581	0.60589
% k-CAR	−0.09664	0.6238	0.03675
